# Exploring attitudes to research involving human subjects among Vietnamese university students: establishing a prospective longitudinal mixed-methods student cohort at the University of Medicine and Pharmacy at Ho Chi Minh City

**DOI:** 10.12688/wellcomeopenres.19632.2

**Published:** 2024-06-19

**Authors:** Vy Pham -Tram, Nguyet Minh Nguyen, Susan Heatherley, Kien Vu Duy, Bao Hung Vu, Giang Pham Thi Huong, Hang Nguyen Thi Thu, Hong Huynh Thuy Phuong, Truong Thi Tuyet Thanh, Chi Le Phuong, Thuy Nguyen Thi Van, Trung Dinh The, Dong Thi Hoai Tam, Mary Chambers, Katrina Lawson, Jennifer Ilo Van Nuil, Dung Do Van, Tuan Diep Tran, Evelyne Kestelyn, Bridget Wills

**Affiliations:** 1 Oxford University Clinical Research Unit, Hospital for Tropical Diseases, Ho Chi Minh City, Vietnam; 2 University of Medicine and Pharmacy at Ho Chi Minh City, Ho Chi Minh City, Vietnam; 3 Centre for Tropical Medicine and Global Health, University of Oxford, Oxford, UK

**Keywords:** Vietnam, university students, attitudes, human subjects’ research, mixed methods

## Abstract

Research capacity is increasing in low- and middle-income countries (LMICs), with progressive development in the range and complexity of studies being undertaken, often in collaboration with high-income country partners. Although senior local stakeholders are typically involved in ensuring that research is conducted according to accepted standards for ethical and scientific quality, to date there has been little exploration of the views of younger generations around the ethics of research involving human subjects.

We present our protocol to establish a longitudinal mixed-methods student cohort at the University of Medicine and Pharmacy at Ho Chi Minh City, Vietnam, that is investigating students’ views around the ethics of clinical and public-health oriented research. We use a synergistic approach involving initial deliberative engagement activities ( *e.g.* science cafes, debates) to inform participants about complex concepts, prior to formal quantitative and qualitative methods (surveys, focus group discussions and in-depth interviews) that are designed to explore the students’ views in detail. We focus in particular on dengue research, i.e. research that addresses a locally relevant disease with which the students are likely familiar, and probe their thoughts on such themes as appropriate remuneration for research participants, involvement of vulnerable groups, use of human challenge trials in LMICs *etc.*

A snapshot of the cohort and its activities after one year is also presented; among 429 active students, primarily from the Faculty of Medicine, the proportions of male and female students were similar, the majority were from southern or central Vietnam where dengue is endemic, and available data indicates the cohort to be representative of the expected spectrum of socioeconomic groups.

The cohort provides a unique resource to investigate the views of young people on medical ethics, an important but hitherto underrepresented group in such discussions. Feedback indicates a clear interest in contributing thoughts and ideas to the development of clinical research in Vietnam.

## Introduction

At the turn of the millennium the Global Forum for Health Research highlighted the uncomfortable truth that, while low/middle-income countries (LMICs) accounted for 85% of the world’s population and 92% of the global disease burden, less than 10% of global spending on health research was dedicated to overcoming the health challenges prevalent in these countries ^1, 2^. Subsequently the 2013 World Health Report emphasized the need for research evidence to be generated by all nations to achieve the goal of universal health coverage ^3^. There has also been a conscious commitment to improve health in LMICs via the establishment of the United Nations Sustainable Development Goals (SDGs); in particular SDG3 endeavours to “Ensure healthy lives and promote well-being for all at all ages” ^4^. An integral part of this commitment involves the promotion of partnerships between high-income countries (HICs) and LMICs to improve health inequalities by developing health research capacity in LMICs. In recent years there has been a marked increase in the range and complexity of studies undertaken in partnerships involving HIC and LMIC actors, most dramatically following the onset of the coronavirus disease 2019 (COVID-19) pandemic in early 2020 ^5, 6^.

Given the financial and structural inequalities that exist between LMICs and HICs, it is crucial that such partnerships are mutually beneficial and provide long-term health benefits for all partners and communities involved ^7– 9^. Although ethical and regulatory systems to support oversight of such research efforts are regularly reviewed, intermittent reports of unethical or inappropriate practices, together with a general lack of knowledge about clinical research among LMIC populations, can increase public anxiety ^10, 11^. In turn this may contribute to development of anti-science views, potentially exacerbating the vaccine hesitancy that is becoming increasingly apparent globally ^12– 14^. In recent years, increasing recognition of the power imbalances and structural inequalities inherent in transnational partnerships has resulted in renewed efforts to promote inclusivity and social justice. Progress remains slow, but efforts include allocation of research resources directly to partners based in regions with a high burden of the disease of interest, support to develop local infrastructure and expertise, and, most importantly, recognition of the importance of considering the experience and voices of potential participants in relevant communities, at all stages from research design to dissemination of results ^15^.

The Oxford University Clinical Research Unit (OUCRU) is a large-scale Wellcome funded clinical and public health research facility with sites in Vietnam, Indonesia and Nepal. For over 30 years, OUCRU has worked closely with local partners in Vietnam to coordinate a comprehensive programme of research focused on infectious diseases and other locally relevant health issues. All OUCRU research must be performed in accordance with internationally recognized standards for ethical and scientific quality, but the importance of understanding the views and principles of local stakeholders, research collaborators and potential study participants is also recognised.

In the past very few studies have investigated attitudes towards health research among the general public in Vietnam, or explored Vietnamese people’s willingness and motivation to participate in research. One contributing factor could be the relatively low priority accorded to research in the national health agenda in the past, compounded by a lack of resources allocated to support studies examining the perceptions and attitudes of the population, and a general lack of experience with processes designed to obtain input from the community in relation to health research ^16^. The absence of an established and trusted system of dialogue between researchers and the public is one specific factor that was highlighted as likely to impede research efforts, particularly with respect to study enrollment and retention ^16^. A recent study that focused on the process to obtain consent for clinical studies in Ho Chi Minh City (HCMC) also highlights the importance of terminology; for example, the Vietnamese phrase for “research” also means “experimentation”, a term which could invoke fears of being “a guinea pig” or “being the subject of an experiment”, and thus might pose the first barrier to an individual’s motivation to participate when approached ^17^.

Another potential barrier relates to limited health literacy among the Vietnamese public, which could result in contextual challenges for both researchers and participants. Nguyen et al found that patients’ lack of knowledge about their condition, the harmfulness of self-prescribing, and the potential risks of late diagnosis and treatment initiation, contributed to notable delays in accessing effective healthcare for chronic lung disease ^18^. Similarly, Nguyen et al, identified difficulty in understanding or interpreting study information as a significant barrier limiting potential volunteers’ decision to participate in research ^19^. The authors also comment that this “fragmented understanding” is influenced by hierarchical relationships between participants and physicians in Vietnam, and suggest that use of more effective engagement and communication approaches might help to lower these hierarchical hurdles.

For these and many other reasons it is now accepted that understanding the particular socio-political and cultural context, collecting information on the acceptability of research among the population, and enhancing involvement of the public in the research agenda from the outset, are all vital phases that are necessary to promote establishment of a transparent and trustworthy culture in which research can flourish.

One disease of particular importance for Vietnam is dengue, a mosquito-borne viral infection that results in major disease and economic burdens for the country ^20, 21^. Estimates of 2.2 (2010) and 2.6 (2013) million symptomatic dengue cases annually have been suggested for Vietnam by different groups ^22, 23^, far in excess of the officially reported case numbers. Hospitalized patients form only a small minority (less than 20%) of these cases and deaths are rare. However, estimates for the overall health and economic burdens for the country per year are substantial, almost 40,000 DALYs and more than USD 90 million respectively in one assessment based on 2016 prices ^21^. Although Vietnam’s public health insurance program covers a proportion of the direct medical costs for infected individuals, dengue remains financially burdensome for patients and their families due to significant unreimbursed direct costs as well as multiple indirect costs. Meanwhile, disease pathogenesis, virology and immunology are complex and poorly understood ^24^. No specific therapeutics are available and development of safe and effective vaccines is proving difficult ^25^.

Many types of dengue research are undertaken by OUCRU and its partners ( https://www.oucru.org/research/dengue/#undefined), including observational studies, cohort studies, therapeutic intervention trials and human-to-mosquito transmission studies, some of which involve vulnerable populations such as children and/or very sick patients in intensive care units. In addition, novel research approaches, including the potential use of human challenge models, are under consideration. Senior local stakeholders are always involved in discussions and decision-making about OUCRU’s dengue research studies, but as the breadth and range of activities expands there is increasing need for engagement with stakeholders at all levels of society to ensure that an equitable and balanced approach is adopted.

The unique perspective of adolescents and young adults is increasingly being recognised, with the importance of listening to these views and involving them in advocacy roles and policy development highlighted in a recent Lancet editorial ^26^. Although studies have been undertaken in various other countries to explore young people’s views about research ethics ^27– 29^, until recently no such research had been carried out to understand the opinions of younger generations of Vietnamese society about clinical and/or scientific issues related to research. Notably however an on-line survey was conducted during the pandemic to look at predictors of COVID-19 vaccine acceptability among Vietnamese health profession students ^30^.

In late 2019, with funding from Wellcome, we started a programme of work to explore attitudes towards research in humans across a range of different Vietnamese stakeholders, with a project entitled RESHAPE ( RESearch in Humans: Attitudes and PErceptions). Under this umbrella we have developed a prospective longitudinal cohort of university students (the SEED cohort) enrolled at the Faculties of Medicine and Public Health at the University of Medicine and Pharmacy (UMP) at Ho Chi Minh City (HCMC), with the aim of exploring Vietnamese students’ perceptions and views of clinical and public health orientated research. We plan to use the information generated to improve our understanding of public perceptions and possible barriers to acceptance of clinical research in Vietnam from the youth perspective, in order to better design informational material and public engagement activities for future research, especially for novel or complex studies.

Here we present the protocol for the study, describing the approach we used to establish the cohort and the various techniques employed to explore the students’ views. We also present an overview of the structure of the cohort after the first year of activity.

## Methods

In setting up this prospective mixed-methods social science research platform our main goal has been to establish a cohort of university students undertaking their studies in Ho Chi Minh City who are willing to explore attitudes towards clinical and public health orientated research in Vietnam from the youth perspective. Our approach is designed to alternate two main synergistic components to explore a range of different ethics related themes; thus an initial deliberative engagement process intended to educate and inform cohort members about complex concepts is combined with a mixed-methods data collection approach incorporating both quantitative and qualitative methodology ^31^.

Using a mixed-methods data strategy allows the research team to use a range of techniques, combining both inductive and deductive thinking in a complementary approach that maximizes the strengths of each data type to facilitate a more comprehensive understanding of the issues under consideration. From the quantitative perspective, we incorporated serial surveys into the study design, based on our assumption that the attitudes and experiences of the students would likely evolve over the course of three years, and that their engagement with the cohort activities might influence their views. Coupling this quantitative approach with open-ended interviews and focus group discussions allows us to explore various explanations for their views and supplement the data with insights that we might have missed from surveys alone ^31^.

We elected to focus our recruitment among medical and public health students, since it is possible that these individuals may become involved in the execution of clinical research studies in the future, either as junior clinicians or scientists contributing to data collection or laboratory studies, or potentially as future research participants. Secondly, we wished to recruit cohort members early in their university careers and to follow them over several years, to assess how their advancing medical and scientific knowledge and awareness might influence their attitudes over time. For logistic reasons we have recruited first and third year students enrolled at the two faculties at UMP, who then progress through their studies while involved with the SEED project, so that after 3 years the cohort includes individuals at all stages of their undergraduate education across the range of academic disciplines taught at UMP. Thus we are in a position to explore the diversity of perceptions and attitudes of students with different backgrounds, training, levels of knowledge and interest in science and medicine. A brief summary of the structure and basic course content for the various student groups we approached, is provided in the appendix (Table S1).

We are interested to assess socio-cultural factors that may contribute to these views, but rather than targeting students from particular geographical or socioeconomic backgrounds, we aim for broad recruitment across the student body. Although our overall approach is general, where appropriate we focus on dengue-related research, as an example of a specific disease that is of considerable relevance to this population.

### Recruitment of participants to the SEED cohort

With formal agreement from the university governing body, each year promotional flyers are posted on relevant social media sites prior to a series of introductory talks given separately to 1 ^st^ and 3 ^rd ^students immediately after lectures/activities that the student body are expected to attend. The sessions are presented by members of the OUCRU RESHAPE team (comprising clinicians, social scientists, and public engagement and communication personnel), who use a variety of methods including formal presentations, short debates, question and answer sessions, icebreaker activities, and quizzes to increase interaction with students to present information about dengue and different types of clinical research, and also to highlight some of the important ethical and practical considerations involved in such research studies. These talks are intended to raise awareness of the rationale for developing the student cohort and to encourage students to volunteer if they find the ideas interesting but are not part of the standard curriculum. 

After each talk, the students are given an information sheet or a link with an OUCRU contact point, offering an opportunity to formally join the student cohort. Those who express interest are invited to attend small group (10–20 individuals) meetings where more detailed information is presented about the planned activities within the cohort, and those who choose to proceed are invited to sign an Informed Consent Form (ICF). OUCRU has no official standing within the Vietnamese university hierarchy and the information sheet makes it clear that involvement is entirely voluntary.

Each student who joins the cohort is given a unique code which is used to identify them in all subsequent activities. Additionally, each student is asked to complete a demographic questionnaire (see extended data ^32^) describing their background, family structure and socioeconomic status.

### Ensuring effective communication with cohort members

For informational purposes we use the project website ( https://www.reshape.oucru.org) and Zalo, a widely popular texting application in Vietnam. The website serves as the official communication channel, where cohort participants can browse general information about the project, view team member profiles, and learn about our activities. Zalo is utilised for quicker and more direct communication, with different chat groups created for the different academic years; notifications are sent directly to every member in the chat group, so this platform is most suited to send logistic updates and important practical information to the students.

For engagement and general conversation, we created a private Facebook group, operated under the umbrella of OUCRU’s official Facebook page. Only cohort participants may join, so the students can feel comfortable initiating and engaging in discussions with other members as well as the project team. 

Finally, the SEED Ambassadors group helps the project team to connect with the students in a more intimate and friendly way. These ambassadors facilitate active two-way communication between the team and the student body, providing suggestions on how to engage with the students more creatively and effectively, while also acting as representatives of the cohort to raise opinions and give feedback.

### Cohort activities

The first activity for all newly recruited students is a kick-off half-day seminar designed to introduce the students to each other, OUCRU and the study team, and to briefly review the material presented during the introductory talks. Subsequently a variety of important themes relating to clinical research in human subjects are approached sequentially, combining in-depth qualitative methods with synergistic engagement activities (see Appendix Figure S3 for a schematic describing the flow of activities during the first year ^33^). We start with deliberative engagement activities designed to introduce the often-complex ideas related to a particular theme at interactive events (science cafes, science debates); at these events participants are encouraged to explore their thoughts and opinions in a non-threatening space where all ideas are welcomed. Later, after they have attended one of the engagement events related to a specific theme, we invite around 8 – 10 selected students by email, to participate in focus group discussions (FGDs) or in-depth interviews (IDIs) that examine that particular theme in more detail. Individuals (including students and their family members) who participate in any event receive a small sum (<10 USD) to compensate them for their time and effort.

For the first theme we focused on the broad question, “What is clinical research and how is it relevant to Vietnam?”, to encourage students to become familiar with the general approach. To develop the subsequent themes, we initially explored the literature in our particular field of interest, i.e. focusing on complex ethical issues related to involvement of healthy volunteers in clinical research. Based on early feedback from the first engagement activities with the students, we selected the themes the group found most interesting, but also attempted to incorporate any issues raised by cohort members into our evolving plans. The final themes selected for the initial phase covered inclusion of vulnerable groups in clinical studies, how to set appropriate standards for remuneration of research participants, recruitment of healthy volunteers for vaccine trials, and use of human challenge studies in LMIC settings (specifically dengue challenge studies).

A new theme is introduced every 3–4 months, and all students who are cohort members at the time a particular theme is explored are eligible to participate. Each engagement event for a new theme is advertised widely across the various communication channels we operate, and any student who wishes to take part is asked to sign up on-line to express their interest. Staff then select specific students to invite to the activity, the only limitations to participation being the capacity of the venue and the number of research staff available to support a particular event effectively. However, preference is given to students who have not previously attended an activity. We aim to provide each student at least one opportunity for active involvement each year they remain in the cohort.

Science cafés. We employ a modified science café format adapted from the general principles of the world café design ( https://theworldcafe.com) ^34, 35^. Groups of 30–40 students are invited to attend a meeting during which general information on the topic of interest is first presented, incorporating visual aids and/or short games to explain complex concepts, following which groups of 6–8 students are asked to consider a series of questions related to the particular theme. Over about an hour, research team members observe and support, but do not direct, the ensuing conversations, following which student representatives summarise the main points discussed; the students’ points, as well as any field notes recorded by the team, are then used to feed into the discussion guides used during related activities. Small gifts are presented to the student representatives who coordinate these group discussions, as well as prizes for other students considered by the project team to have been particularly proactive.

Science debates. Formal debates are commonly used as pedagogical tools in HICs but remain unfamiliar to students in Vietnam ^36^. The technique can be effective in encouraging individuals to consider different aspects of a problem in order to establish a position and express a viewpoint using supporting arguments and evidence ^37– 40^. Groups of 40–60 interested students are informed in advance of the debate question, receive information on the basic principles and structure of a debate, and are given pointers to relevant on-line resources to explore the available evidence. At a preliminary meeting the students are asked to vote for or against the motion and are then separated into two approximately equal teams, in line with their choice where possible. The students are informed that each team’s overall performance will be assessed in terms of style, strategy and content, and particular study staff make themselves available to the opposing groups to support them with background research. Subsequently, during the actual event, an experienced external speaker reiterates a summary of the general structure and rules for the activity, and the two sides are given 50 minutes to prepare together before nominating four representatives for the formal debate. The speakers from each team alternate their arguments for or against the proposition, with the final speakers summarising their team’s overall position, after which a general discussion takes place before the student body votes again on the motion. 

An adjudication committee, comprised of the external speaker and two senior SEED project staff, rates the overall performance of the opposing teams and provides feedback to the students on their efforts before awarding the committee prize to the winning team. In addition, a “people’s choice award” is presented to the team favoured by the student body. 

Focus group discussions and in-depth interviews. Following on from the engagement activities centred around a particular theme, students are purposefully selected to cover a range of socioeconomic backgrounds and are invited to attend either an FGD or an IDI. The FGDs are suited to gathering rich and diverse perspectives, stimulated by interactions between the students present, while the IDIs are employed later to cover more sensitive information or intricate themes in detail. Family FGDs are also arranged, involving small groups of students each accompanied by a chosen family member. At these events we investigate the role of parents in students’ decision-making. 

Guides for the FGDs and IDIs are developed after reviewing the published literature around that theme and taking into account issues raised by the students themselves during the relevant engagement activities. The guides concentrate on questions related to perceptions of and assumptions around important concepts related to the theme, as well as practical aspects of different types of research. Additional topics and probes are included as necessary during the events, depending on the nature of the evolving discussion.

Student-led participatory initiatives. In addition to the events listed above, we also incorporate student-led approaches in our engagement agenda. As an example, cohort members are intermittently offered opportunities to conduct their own fieldwork, by interviewing their peers or family members to explore perceptions and attitudes related to SEED project topics of personal interest to themselves. Following appropriate training the students are encouraged to take the lead in developing ideas for these conversations, and then to use their mobile phones to record interviews and upload a copy of the unedited material to the OUCRU secure server for review and assessment by project staff. Subsequently, some students are selected from among those submitting video interviews (based on the length, technical quality and content of their submission), and trained to make more complex short films that are designed to be shown publicly.

Another opportunity involves creation of scientific posters or short (2–3 minute) informational videos that focus on topics relevant to the project. Interested students are invited to attend a training workshop where they receive guidance on how to develop informative content and select creative and effective communication methods. The students are then encouraged to develop their own material, working either as individuals or together in small groups, with SEED team members providing advice and support as necessary. The resulting posters and videos are then showcased at an exhibition held at UMP, that aims to raise awareness of the SEED cohort within the wider student population. A panel of judges selects winning entries in different categories and prizes are presented during the event; subsequently, the posters and videos are also exhibited in a public space within the university, thereby encouraging wider dissemination of the students’ ideas.

The experience of taking the lead in the research cycle, from developing an initial idea through to execution of all the necessary activities required to deliver an output suitable for public display, has proved very popular with the students ^41^. These initiatives also encourage the bi-directional flow of thoughts and ideas between SEED project staff and cohort members, ensuring the study team’s input is responsive and supportive rather than unidirectional and reinforcing our efforts to ensure the students understand that their knowledge and capabilities are respected and valued, all of which are important principles of participatory research ^42^. There is no monetary benefit for these student-led participatory initiatives. However, comprehensive skills training is provided for those wishing to take part, and all students who submit an entry also receive a small gift of appreciation and a certificate of recognition for their participation.

### Ethical approvals and informed consent

The research programme follows international standards for the ethical conduct of research involving human subjects, and all staff involved have up-to-date training in Good Research Practice (GRP) ^43^. The protocol and related documents have been reviewed and approved by the Ethics Committee at UMP (Reference number: 351/UMP-BOARD approved on 26 May 2020) and the Oxford Tropical Research Ethics Committee (OxTREC Reference: 540-20 approved on 02 July 2020). 

All participants, who must be aged 18 years or more, are provided with a detailed Participant Information Sheet (PIS) before signing the ICF. Cohort members are asked at intervals if they would consider inviting their parents/adult family members to certain activities; if so, the students discuss the study with their relatives and provide them with a copy of the PIS/ICF to read. Relatives who wish to participate contact the study coordinator to register their interest, who then invites them to attend relevant family FGDs or IDIs as they occur. Staff review the PIS/ICF with family members before an activity commences, and all present are asked to sign a consent form.

Specifically with respect to video or filmmaking activities, verbal consent is recorded on camera at the start of the video/film, in accordance with the protocol approved by the Ethics Committees at UMP and OxTREC. Since visual data collected by the students could reveal the identity of individuals involved, students receive training in GRP before participating in any collaborative data collection. The initial verbal consent is an agreement to be interviewed and filmed by the students, with the understanding that any resulting material is intended only for internal use among the study participants and researchers. However, possible wider uses of the data are discussed with all participants prior to filming, and they are asked specifically if they would permit their data to be used for public activities. Subsequently if any material is selected to be made public, a draft of the final product is sent to all individuals involved in that particular video/film, so that they may decide if they wish their contribution to be included; at this time they are asked to provide written informed consent.

### Data collection and analysis

Both quantitative and qualitative data collection methods are employed. As well as the demographic questionnaire, all students are asked to complete a Comprehensive Survey (see extended data ^32^) designed to explore their general perceptions of and attitudes towards clinical research, when they attend their first event and again at serial time points during the life of the project to explore changes over time. To develop this tool, we carried out translation and back translation, as well as pilot testing and cognitive interviews with representative students to check content validity and ensure the final Vietnamese wording was appropriate.

We use standard statistical methodology to describe the general characteristics of the cohort population. We aim to engage widely with students and their families, to ensure broad representation across the spectrum of socio-economic backgrounds. The approaches used to classify the socio-economic status of each family, and the occupational categories of the parents/guardians, using information provided in the demographic questionnaire are described in the extended data ^32^. In due course, we will explore how these demographic and socioeconomic characteristics influence students’ awareness of and attitudes towards research and research ethics. We also plan to explore potential effects of other factors such the level of participation in cohort activities, the influence of a student’s UMP Faculty and/or Department etc. Additionally, among students who complete the Comprehensive Survey more than once during their period of involvement with the SEED project, we will be able to assess how their advancing medical and scientific knowledge influences their attitudes to research ethics over time.

Information from field notes, audio recordings and video files are transcribed into electronic word documents, using only the unique identifying code assigned to each participant at recruitment. Information from the FGDs and IDIs of cohort participants and their family members is then translated as necessary, and the data uploaded into Nvivo 12 for management and coding. Alternatively, the free Coding Analysis Toolkit (CAT) is another option that can be utilized for performing comparable tasks. For the qualitative research component, we use a grounded theory approach ^44^ employing alternating cycles of data collection, interpretation and analysis ^45, 46^. First, we identify recurring ideas and concepts from the initial coding of data and generate a preliminary codebook. This is then used to pose emergent enquiries that require further exploration; following adaptation of the interview discussion guides, additional data is gathered through a second round of in-depth interviews with more students. Finally, we adjust and combine all the coding with more field data and further review of the literature and discuss the emerging themes until the final codebook is established. 

### Confidentiality and data protection

Participants are assured that all information generated, including all audio and visual digital media, will remain confidential and be securely stored in accordance with OUCRU policies.

Quantitative information is collected primarily via on-line questionnaires and surveys using the JISC Online Survey platform. Upon completion of any survey the data are exported promptly to the OUCRU secure server. The qualitative data takes the form of written field notes and audio/video files used for recording the various activities. All data for which there is no clear consent are destroyed, while audio and video files obtained with appropriate consent are transferred to the OUCRU secure server. Ownership of all images and videos collected by the cohort participants on their own devices remains with the students themselves; their responsibilities with respect to confidentiality are highlighted on the PIS/ICF that each student signs at the start of the study. All material recorded on project equipment belongs to the project and the students may not keep copies themselves unless the material is in the public domain.

Original essential documents and any audio/video files that include informed consent are maintained for a minimum of three years after the last interaction with any of the cohort participants. Subsequently these documents, together with all other materials generated from project activities, will be stored in an electronic archive on the secure OUCRU server for an additional period of 10 years.

### Adaptations to study procedures related to COVID-19

As a result of the COVID-19 pandemic, all activities were transferred from in-person to on-line formats from June 2021 until March 2022. Activities are carried out in compliance with local government regulations, UMPs planned teaching schedule ( *i.e.* in-person *versus* on-line lectures), and the OUCRU policies for health and safety in place at any particular time.

The format for the on-line activities remains very similar to the in-person events but conducted via a web platform making use of break-out rooms and digital notice boards and supported by an active social media presence. To minimise social anxiety and encourage a collegiate atmosphere, participating students are sent a colourful project backdrop beforehand. Support staff are also on hand to deal with any technical issues and to encourage participants to feel comfortable in the on-line space so that they are empowered to express their opinions. 

### Timelines and study status

From May to July 2020, we obtained the necessary ethical approvals to set up the cohort and developed relevant materials for the initial engagement activities. The first introductory talks and enrolment events took place in July 2020, resulting in recruitment of some 200 students. Subsequent recruitment waves have occurred at intervals up to December 2021, typically in conjunction with the annual new student intakes at UMP.

Cohort activities started in October 2020 and take place intermittently throughout the university calendar year, with novel themes introduced every few months. The engagement activities and in-depth social science qualitative methods are synergistic with each other; events related to a particular theme are scheduled to occur in parallel so that findings from the engagement work can inform the social science data collection and vice versa. The themes previously highlighted in the Methods section were covered sequentially during the first 2 years of the project, culminating with a major theme focusing on use of human challenge methodology in LMICs during the latter half of 2022. For the current work (2023), we are exploring cross-cultural differences in perceptions and attitudes to human challenge studies among comparable groups of students recruited from different countries.

In this manuscript, we present a detailed summary of the study design and methodology used to recruit and maintain the Vietnamese cohort, alongside a comprehensive description of the practical aspects of the various engagement and social science activities undertaken. We are presently analysing the qualitative and quantitative data generated around particular themes of interest and are using the findings to explore several important research questions. By providing a clear methodological description of the cohort and its complex activities, we hope to facilitate understanding and interpretation of our findings as they are published. On overview of the general structure of the cohort at one year is presented in the next section.

## Overview of the cohort structure at one year

**Figure 1.**
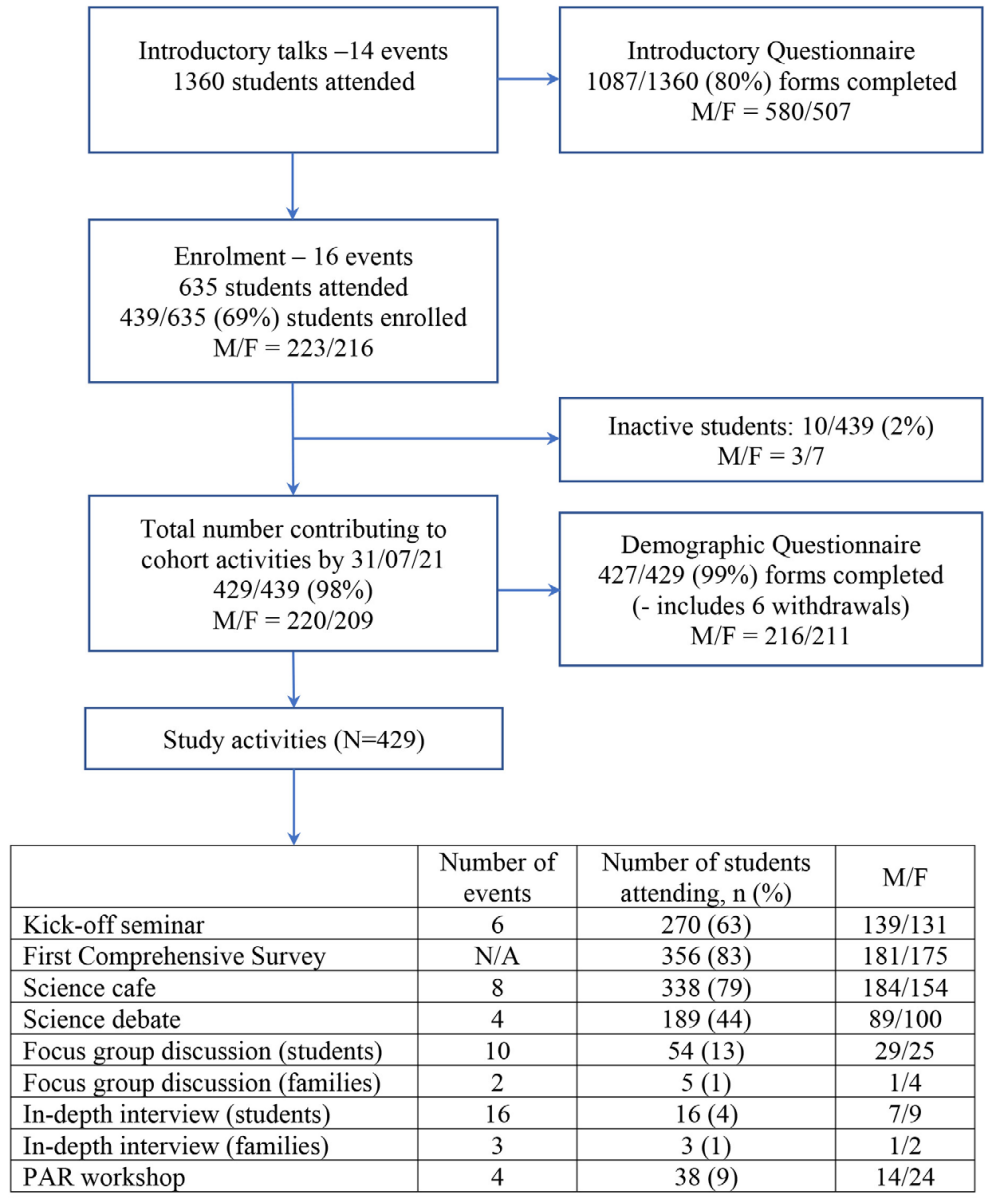
Flow chart of recruitment numbers and cohort activities up to 31st July 2021.

Although the cohort continues to evolve, for the purposes of this manuscript we concentrate on describing the general structure as of 31st July 2021. At this time, 1360 students had attended at least one introductory talk and a total of 439 students had consented to join the cohort ( Figure 1). Among the 439 students enrolled, 10 had neither completed a questionnaire nor attended any events (inactive students). An additional six students had formally withdrawn citing “time pressure”, but all six consented for their data to the point of withdrawal to be retained for analysis. A total of 52 activities (either offline or online) were organised during the first year, with 370/429 (86%) active students attending at least one event.

Summary demographic information for 427/429 (99%) of these active students is presented in Table 1. Most participants were from the Medical (71%) rather than the Public Health (29%) Faculty. The majority were representative in age of their university year-group (18–22), with only two students being older than 23, (25 and 29). The overall proportion of male and female students who enrolled was similar, in line with the demographics for UMP admissions in the relevant years. Subsequent involvement in the various cohort activities was also reasonably balanced between male and female students. Most students (379/424, 90%) were ethnic Kinh, the most common ethnicity in Vietnam, with only a few other Vietnamese ethnic minority groups represented, plus a few individuals of Cambodian Khmer and one of Thai descent. Students’ family sizes were generally small. In most instances at least one parent/guardian had completed higher/tertiary education (61%) and in 148/402 (37%) of the families for whom classification was possible, at least one parent/guardian was employed in a managerial/professional capacity.

**Table 1.** Overview of the demographic characteristics of students in the cohort on 31 ^st^ July 2021, stratified by their academic year at enrolment.

	N	First year (n=204)	N	Third year (n=223)
**Age**	203	18 (18 – 19)	222	21 (20 – 21)
**Gender - male**	204	101 (50)	223	115 (52)
**Year enrolled at UMP**	204	- 2019: 22 (11) - 2020: 182 (89)	223	- 2017: 56 (25) - 2018: 167 (75)
**Department ^$^** - Medicine - Preventive Medicine - Public Health - Nutrition	204	- 139 (68) - 22 (11) - 25 (12) - 18 (9)	223	- 163 (73) - 34 (15) - 18 (8) - 8 (4)
**Ethnicity** - Kinh - Hoa - Khmer - Other	203	- 182 (90) - 13 (6) - 4 (2) - 4 (2)	221	- 197 (89) - 11 (5) - 5 (2) - 8 (4)
**Family size** - Adults living in family home - Children living in family home	199	- 2 (2 – 3) - 1 (0 – 1)	222	- 3 (2 – 4) - 0 (0 – 1)
Educational level of parent/guardian * - Primary education - Secondary education - Higher/tertiary education	170	- 16 (9) - 46 (27) - 108 (64)	203	- 31 (15) - 52 (26) - 120 (59)
Occupational category of parent/guardian * ^&^ - Managerial/professional - Technical and clerical support, service and sales - Skilled workers - Unskilled workers - Armed forces - Unknown	204	- 66 (32) - 68 (20) - 34 (12) - 16 (7) - 3 (1) - 17 (8)	223	- 82 (37) - 71 (32) - 47 (21) - 10 (4) - 5 (2) - 8 (4)

Data are presented as median (interquartile range) for continuous variables and number (percentage) for categorical variables. Note that 2 of the 429 students enrolled in the cohort by the 31 ^st^ July 2021 did not complete the demographic questionnaire. ^$^: The Public Health Faculty includes the departments of preventive medicine, public health and nutrition *: For each family, the highest attainment for either parent (or a guardian) is presented ^&^: Occupational status was classified in line with the International Labour Organization’s recommended system, ISCO-08 (see extended data for details) ^32, 47^.

**Figure 2.**
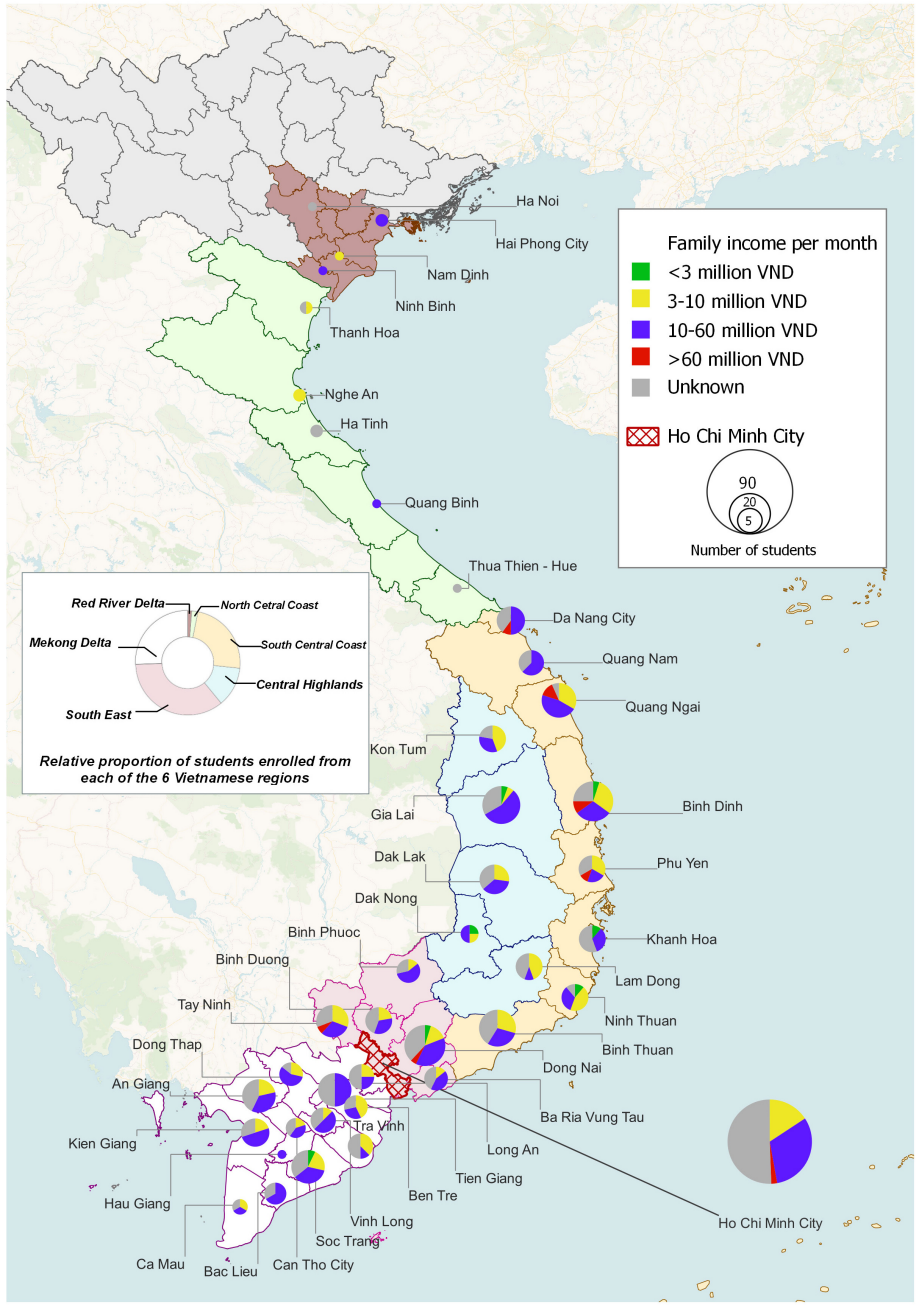
Distribution of students’ home addresses across Vietnam. The map depicts the distribution of students’ home addresses across Vietnam, at province level, for the 412/429 (96%) of students who enrolled during the first year of the cohort and declared this information. The students’ personal assessments of their families’ monthly income in Vietnamese Dong is also presented, as a proxy for socio-economic status. Note that only 256/412 (62%) of the students provided information in this domain.

Figure 2 presents information on the provinces across Vietnam from which 412/429 (96%) of the students originated ( *i.e.* the family home location), plus an assessment of socioeconomic status for the students who provided information in this domain. A majority had been brought up in provinces in southern or central Vietnam, with relatively few from Northern provinces. Around 35% were from the South-East region (which includes HCMC), with another 25% coming from the Mekong Delta and 25% from the South-Central Coastal region.

A total of 256 students (60% of the cohort) provided information on socio-economic status, and among this group almost all families fell within the low-normal or high-normal categories (see extended data for definitions ^32^); only 8/256 (3%) came from “poor’ families and 10/256 (4%) from “wealthy” families. A “poor” family background was most evident among students from the North/South-Central Coastal areas or from the Central Mountains, where the regional poverty rate is reported to be 1.5 to 2 times higher than the national average ^48, 49^. “Wealthy” families were also identified in the Central Coastal provinces, as well as in the South-East around HCMC, in keeping with published literature indicating these areas as among the most affluent in Vietnam ^50^.

## Discussion

Here we have outlined the methods used to develop a large social-science-oriented student cohort at the University of Medicine and Pharmacy at Ho Chi Minh City, which aims to investigate students’ views of clinical research in human subjects and have described the general structure of the cohort after one year. Although efforts to explore students’ opinions on various aspects of research ethics are becoming more common, most studies have been cross-sectional and have focused on specific topics ^51– 53^, and often have involved only small numbers of participants ^54– 56^. To our knowledge this is the first time this approach, *i.e.* exploring the youth viewpoint on a range of complex ideas related to clinical research among a large number of university students and specifically incorporating a longitudinal perspective, has been employed.

We hoped to include a broadly representative group of Vietnamese students with an interest in biomedical research, ideally engaging with individuals from a wide spectrum of geographic locations and social backgrounds across the country. Although UMP has a broad recruitment and admissions policy, representation from northern Vietnam has proved to be limited, probably reflecting the fact that many students from the north choose to attend universities that are closer to home. However, the cohort does include members from all 37 provinces across southern and central Vietnam. This is relevant given our particular focus on dengue research. Dengue is hyperendemic in southern Vietnam, and common in the central region, so it is likely that some cohort members will have had personal experience of dengue within their circle of family and friends, that may shape their knowledge and attitudes. Analysis of the accumulating data will take such prior exposure into account, potentially providing interesting insights into the effects of personal experience on the views expressed by students.

Identifying socioeconomic status was complex; many students were unaware of the precise details of their parents’ educational background and income or were unwilling to disclose this information. It is common across many cultures to be guarded about revealing personal financial information ^57^. Additionally, many Vietnamese families engage in informal small-medium business activities alongside regular employment so it might be difficult for students to assess their families’ overall income ^58^. Although socioeconomic determinants clearly influence many aspects of an individual’s life (including access to higher education in Vietnam ^59^, determining appropriate ways to assess socioeconomic status in a particular context is not straightforward ^60, 61^. However, although use of poorly defined, potentially inadequate, indicators of socioeconomic status may influence research findings ^62, 63^, such data can still be useful to describe a research population and ensure that different community groups are well represented ^60^. In our study, for the 58% of the cohort from whom we obtained some data allowing us to infer socioeconomic status, the relative proportions of the different strata were in keeping with what might be expected in different parts of the country. Thus, while recognizing the limitations of the socioeconomic assessment we suggest that participants in our FGDs and IDIs (selected on this basis) are broadly representative of the socioeconomic distribution of the cohort.

However, it is also important to note that most cohort participants were raised in households with a background of high educational achievement and/or professional careers among the parents/guardians. This pattern is not typical of current Vietnamese society, where only 13% of Vietnamese adults have attended tertiary education ^64^. Since young people often follow the career paths of their parents ^65^, and since we are approaching students enrolled in courses at a prestigious university in HCMC with high educational entrance requirements, these findings are not unexpected. It is likely that some of these students may become Vietnamese thought leaders of the future so exploring and understanding their developing views on clinical research and research ethics is valuable in itself; however, we recognize that the findings may not be applicable among young people generally across the country.

Given that many of the concepts proposed for discussion with the student body are complex and/or novel, and that cohort members are recruited from different year groups and from a range of medical and public health disciplines, we elected to use an approach relying on deliberative engagement prior to our FGDs and IDIs. This is a relatively new technique designed to educate and inform participants in a balanced non-directed way about a particular topic ^66^, thereby laying the groundwork for use of formal qualitative data-collection methods to explore participants’ thoughts and ideas more effectively later – *i.e.* students attending the FGD/IDIs are already relatively familiar with the primary themes and are thus more likely to be prepared to talk about their own views on complex issues during the interviews. Although public engagement is increasingly being employed to improve acceptability and implementation of innovative scientific and health-related research efforts ^67– 69^, as well as to support introduction of new public health and/or disease prevention strategies ^70– 72^, deliberative techniques also enable the public to be involved in shaping novel research programmes and potentially in contributing to shared decision-making about what is appropriate in a particular context. Since the information presented in the engagement activities may bias subsequent discussions, we take care to present both positive and negative aspects of each topic, and also encourage the attendees to review a range of literature and audio-visual material covering the full spectrum of views on the topic before attending an FGD or IDI. Overall, the deliberative paradigm helps to overcome knowledge gaps, improves data quality, and encourages participants to become more involved in the conversation ^66, 73– 75^.

From the outset we recognized that developing practical and efficient communication pathways with cohort members would be essential to the success of the project. The various methods we employed were quite effective during the initial phase, and were subsequently augmented by positive reviews from the ambassadors’ group as the project became more established. Later, when all of our activities had to move to an online platform, the Facebook group proved to be an ideal tool to interact with students in a meaningful way. Utilising different features of Facebook (comment, share, polls), we created various campaigns and activities to keep students engaged and interested in the project despite an extended period of social distancing, successfully maintaining the 2-way relationship between students and project staff during this time.

Various adaptations were made to facilitate delivery of the cohort activities virtually, including holding preparatory online meetings to explain procedures and deliver background material prior to big engagement events, use of break-out rooms to provide additional virtual space for group discussion during events, ensuring immediate access to technical support for attendees via a live chat-box *etc*. In addition to COVID risk reduction, online participation saved the students’ time travelling to event venues, and offered opportunities to more students to contribute their thoughts, but some individuals clearly found it uncomfortable to express their views on camera, and technical issues related to internet speeds and/or incompatible equipment frequently occurred. Despite these shortcomings, attendance was typically high at these events and the feedback remained generally very positive. However, both the research team and the students recognized that the vibe, inclusivity and liveliness of the interactions between attendees during in-person events could not be maintained to the same degree during virtual events, and the return to in-person events in recent months has been generally welcomed.

## Summary

A large mixed-methods cohort study has been established in HCMC, Vietnam, designed to explore the attitudes and perceptions of university students towards a range of important themes relevant to clinical research and medical ethics. The student cohort provides a unique resource to investigate the views of this important but hitherto underrepresented group in Vietnamese society; we anticipate that the ensuing reports will provide rich and rewarding insights from the perspective of young people, as well as encouraging continued civic engagement among the participants. Feedback from the students themselves indicates an overwhelmingly positive response to the cohort activities and a clear interest in contributing thoughts and ideas to the ongoing development of clinical research in Vietnam.

## Data Availability

Oxford University Research Archive: Demographic data generated from **“Establishing a mixed-methods student cohort in Vietnam”**, https://doi.org/10.5287/ora-r5b8py5aa ^32^. The project contains the following underlying data: Demographic_Data+2023.03.27.xlsx Oxford University Research Archive: Oxford University Clinical Research Unit **“Establishing a mixed-methods student cohort in Vietnam”** supporting documents, https://doi.org/10.5287/ora-r5b8py5aa ^32^. The project contains the following extended data: Extended Data_Demographic Questionnaire Extended Data_Comprehensive Questionnaire Extended Data_Classification of Occupational Status Extended Data_ Assessment of Socioeconomic Status Oxford University Research Archive: SRQR checklist for **“Establishing a mixed-methods student cohort in Vietnam”**, https://doi.org/10.5287/ora-r5b8py5aa ^32^. Data are available under the terms of the Creative Commons Attribution 4.0 International license (CC-BY 4.0).
