# Supplementary material for: Exploring attitudes to research involving human subjects among Vietnamese university students: establishing a prospective longitudinal mixed-methods student cohort at the University of Medicine and Pharmacy at Ho Chi Minh City
Source: Wellcome Open Res. 2024 Jun 19;8:473. Originally published 2023 Oct 18. [Version 2] doi: 10.12688/wellcomeopenres.19632.2 (PMC11303948; doi:10.12688/wellcomeopenres.19632.2)
Supplement: Peer review history

# Reviewer report: Reviewer response for version 2

**DOI:** 10.21956/wellcomeopenres.23657.r87202

**Published:** 2024-08-12

**Authors:** John Noel Viana

**Affiliations:** 1 Justice and Technoscience Laboratory (JusTech), School of Regulation and Global Governance (RegNet), College of Asia & the Pacific, Australian National University, Canberra, Australian Capital Territory, Australia

The authors have extensively addressed the feedback that I and the other reviewer gave. I am satisfied with the changes they have made.

 I just have a few additional comments regarding Figure 1 and Table 1. For The table in Figure 1, it would be great if the authors could add another column that presents the average number (and range) of students participating in each group activity (kick-off seminar, science café, etc.). For Table 1, I am wondering why the authors presented data on the distribution of participants’ age and family size as range. I think it would be good to present the percentages (of those who are 18 or 19, of those with two or three adults living at home, and of those where there are no children or there is one other child living at home), similar to how information on other variables is presented.

 It might also just be from my end, but I can’t seem to see or access Supplementary Table S1 and Supplementary Figure S3.

 Overall, I congratulate the authors for this exciting project, and I am greatly looking forward to its results.

Is the study design appropriate for the research question?

Yes

Is the rationale for, and objectives of, the study clearly described?

Yes

Are sufficient details of the methods provided to allow replication by others?

Partly

Are the datasets clearly presented in a useable and accessible format?

Yes

Reviewer Expertise:

bioethics, health equity, social studies of science

I confirm that I have read this submission and believe that I have an appropriate level of expertise to confirm that it is of an acceptable scientific standard.

# Reviewer report: Reviewer response for version 2

**DOI:** 10.21956/wellcomeopenres.23657.r89346

**Published:** 2024-08-06

**Authors:** Ryan Spellecy

**Affiliations:** 1 Medical College of Wisconsin, Milwaukee, Wisconsin, USA

This is an interesting and novel article, and the authors have responded appripriately to the prior comments in a manner that helps place the article in the literature.  I have no additional suggestions.  There have been a number of recent articles describing the perceptions of research from numerous stakeholders in LMICs, and this article provides a useful additon.

Is the study design appropriate for the research question?

Yes

Is the rationale for, and objectives of, the study clearly described?

Yes

Are sufficient details of the methods provided to allow replication by others?

Yes

Are the datasets clearly presented in a useable and accessible format?

Yes

Reviewer Expertise:

Research ethics, informed consent, IRBs, and bioethics generally.

I confirm that I have read this submission and believe that I have an appropriate level of expertise to confirm that it is of an acceptable scientific standard.

# Reviewer report: Reviewer response for version 2

**DOI:** 10.21956/wellcomeopenres.23657.r87203

**Published:** 2024-07-11

**Authors:** Suryane S Susanti

**Affiliations:** 1 Universitas Indonesia, Depok, West Java, Indonesia

I have no further comments to make.

Is the study design appropriate for the research question?

Yes

Is the rationale for, and objectives of, the study clearly described?

No

Are sufficient details of the methods provided to allow replication by others?

Partly

Are the datasets clearly presented in a useable and accessible format?

Yes

Reviewer Expertise:

(maternal health, women health, family and community health, disaster nursing, disability study and spiritual care)

I confirm that I have read this submission and believe that I have an appropriate level of expertise to confirm that it is of an acceptable scientific standard.

# Reviewer report: Reviewer response for version 1

**DOI:** 10.21956/wellcomeopenres.21748.r71613

**Published:** 2024-02-23

**Authors:** Suryane S Susanti

**Affiliations:** 1 Universitas Indonesia, Depok, West Java, Indonesia

**Abstract:**

 This is very good abstract which has already comprises study background, aims/objectives, methods, results and conclusion. It would be more complete if the authors provide more information on the rational of the study which related to aim and objectives.

**Background**:

 It is a very interesting and important subject where the authors have provided related evidence based. However, the background section is less justification on why it focuses on exploring attitudes and the methods used. I suggest therefore to rephrase the background section to focus on this justification of this study which lead to the study aims and objectives.

**Methods**:

 The authors should elaborate more on the qualitative  method. How does the mix method works?  Provide more information on how the cohort study works with mix method.

**Discussion**:

 Within the discussion chapter the author should not only describe the findings and when comparing with other study should also include the reason why it is different from the previous study. What make this study different from the previous similar studies? Would be useful if the authors could explain any research gap identified within the study?

 Overall this is an interesting and adequate study

Is the study design appropriate for the research question?

Yes

Is the rationale for, and objectives of, the study clearly described?

No

Are sufficient details of the methods provided to allow replication by others?

Partly

Are the datasets clearly presented in a useable and accessible format?

Yes

Reviewer Expertise:

(maternal health, women health, family and community health, disaster nursing, disability study and spiritual care)

I confirm that I have read this submission and believe that I have an appropriate level of expertise to confirm that it is of an acceptable scientific standard, however I have significant reservations, as outlined above.

# Response

**Published:** 2024-04-03

**Authors:** Vy Pham-Tram

**Affiliations:** Oxford University Clinical Trial Unit, University of Oxford, Ho Chi Minh City, Vietnam

Thank you for your positive feedback and constructive comments on this article. Below, we address each point of the comment report with our responses formatted in *italics* font. We also made adjusments to our new manuscript version with regards to some of your suggestions.  

**1. Abstract:**

*Unfortunately, due to the word count limit we are not able to add further details to the abstract. However, in the Background section we have provided additional details linking the rationale for the study to the aims and objectives – see below. *  

**2. Background:**

*Thank you for the comment. We have expanded the Introduction to provide better linkage with the study aims, including presenting more information on why understanding attitudes is important. We have also expanded the section discussing the ethical barriers, as per the comment from Reviewer #1.*

**3. Methods:**

*Thank you for this suggestion. We have added more detail in the Methods section to describe the rationale for a mixed-methods approach to data collection, as well as to provide clearer information on how we carried out the data collection in practice.  We have also clarified our overall approach to the themed interactions with the student cohort members, i.e. we highlight the concept of integrating the two main components (deliberative engagement and mixed-methods data collection) to operate synergistically to explore major ethical themes. *  

**4. Discussion:**

*Actually, although some reports are emerging that describe cross-sectional or disease-focused studies which specifically explore ethical issues in Vietnamese populations (details now incorporated into the Introduction section), to our knowledge there have never been any longitudinal ethics studies conducted in Vietnam nor any that focus on the youth perspective. We comment on this in the Discussion, and, since comparison with previous studies is not possible, we focus instead on discussing the importance of the study and review some of the challenges encountered during the first year of implementation.* *The intention in this paper is to describe the methodology we used to establish the cohort and the techniques we employed to explore the students’ views, and also to present a big-picture overview of the structure of the cohort after one year.  In subsequent papers we will describe our findings in relation to particular ethical concepts/questions, and at that time we will be in a position to highlight differences from previous work, comment on any research gaps we identified etc.*

# Reviewer report: Reviewer response for version 1

**DOI:** 10.21956/wellcomeopenres.21748.r69045

**Published:** 2023-11-21

**Authors:** John Noel Viana

**Affiliations:** 1 Justice and Technoscience Laboratory (JusTech), School of Regulation and Global Governance (RegNet), College of Asia & the Pacific, Australian National University, Canberra, Australian Capital Territory, Australia

The paper presents the protocol for and first-year progress of a mixed-methods study that explores the attitudes of UMP students on dengue clinical research. Overall, I find the study really interesting and important, given the under-representation of Southeast Asian countries in ethics research. I support the publication of this protocol and study update; however, the authors can make several revisions to further improve the quality of their paper.

 First, the abstract should specify that the project is on students’ views on dengue research (and not just on dengue, in general).

 In the Introduction, the authors could include some statistics on the prevalence and economic impact of dengue in Vietnam. They could also provide more information on the specific types of dengue research, especially the ones supported by OUCRU, in Vietnam and the ethical/societal issues associated with these types of research, as discussed in other contexts/countries. It would also be great if the authors could include additional information on general views of Vietnamese people towards health/medical research; or if this is not available, relevant research on views of people in other Southeast Asian countries.

 There are also additional details that the authors could include in their Methods section. They need to explain why they chose to recruit 1 ^st^ and 3 ^rd^ year students, in addition to providing a brief background on the four degrees that are covered (Medicine, Preventive Medicine. Public Health, and Nutrition). The authors also need to describe the themes that they intend to explore in the study, and how were these themes identified. Are the authors just planning to discuss ethical issues in research, or are they also planning to cover issues associated with partnerships between high and middle/low-income countries, given that this was extensively described in the Introduction? It is also a bit unclear who will be interviewed, recruited to participate in a focus group discussion, and/or surveyed. Does each student only have to participate in the kick-off seminar, and they are then free to choose which cohort activity to join? Or are there cohort activities that are required for all participants, especially if the study aims to determine changes in attitudes towards clinical research from participation in different activities? Will the authors be comparing the impact of different kinds of activities on students’ attitudes towards/awareness of issues in clinical research? The authors also need to describe if they have a target number of initial interviewees, focus group discussions, and number of participants in a focus group discussion. The authors also need to indicate whether family members that will be interviewed and non-students involved in participatory initiatives will be remunerated.

 The section on Study Status should directly include some information on what counts as a “poor” or “wealthy” family. Figure 1 can also be made more comprehensive by adding a column to the left (or boxes) that indicate when (month and year) each phase was conducted. If possible, the flowchart could also include a section indicating planned future activities (after July 31) to help readers visualize the entire project.

 In the Discussion, the authors could describe how socio-economic status, educational level of family, ethnicity, and/or home address could impact/influence attitudes towards health/clinical research. They might also briefly touch upon how professional training (public health vs. medicine vs. nutrition) can influence understanding of ethical/societal issues in research.

 Finally, the authors might consider double checking their punctuation, especially comma use, for consistency. For instance, a comma should be placed after “of the millennium” in the first sentence of the Introduction. Moreover, “One disease of particular importance” can be replaced with “One disease of particular concern”.

Is the study design appropriate for the research question?

Yes

Is the rationale for, and objectives of, the study clearly described?

Yes

Are sufficient details of the methods provided to allow replication by others?

Partly

Are the datasets clearly presented in a useable and accessible format?

Yes

Reviewer Expertise:

bioethics, health equity, social studies of science

I confirm that I have read this submission and believe that I have an appropriate level of expertise to confirm that it is of an acceptable scientific standard, however I have significant reservations, as outlined above.

# Response

**Published:** 2024-04-03

**Authors:** Vy Pham-Tram

**Affiliations:** Oxford University Clinical Trial Unit, University of Oxford, Ho Chi Minh City, Vietnam

Thank you for your feedback on this article. Here, we'd like to go over each point in the reviewer's report. We will quote the reviewer's comment and provide a response directly below it. 1. "First, the abstract should specify that the project is on students’ views on dengue research (and not just on dengue, in general)." *>> Thank you for pointing this out. We have amended this in the abstract. * 2. "In the Introduction, the authors could include some statistics [.......] if this is not available, relevant research on views of people in other Southeast Asian countries." *>> The introduction has been revised and additional text has been incorporated to address these points. * 3. "There are also additional details that the authors could include in their Methods section. They need to explain why they chose to recruit 1st and 3rd year students, in addition to providing a brief background on the four degrees that are covered (Medicine, Preventive Medicine. Public Health, and Nutrition)." *>> More information has been added to the paragraph describing why we recruited students early in their academic careers. Essentially, by recruiting first and third years studying at the medical and public health faculties at UMP and following them over time, after 3 years the cohort including representatives in each academic year studying across the various courses available at UMP.  (To approach all the students in all the academic years across both faculties would have involved thousands of students, since UMP admits in excess of 700 students to its various courses each year – this would have been impossible for our team both logistically and financially.)* *A short summary of relevant information describing the various degrees offered at UMP is provided in the appendix, since including this level of detail in the main text might distract readers from the main content of the manuscript. * 4. "The authors also need to describe the themes that they intend to explore [....] issues associated with partnerships between high and middle/low-income countries, given that this was extensively described in the Introduction? *>> We have added a short explanation on how the initial themes were selected. As we expected, the structure and quality of research partnerships between HIC and LMIC actors has figured prominently in the students views on the ethics of human subjects’ research in the Vietnamese context. However, the primary purpose of this paper is to describe the methods we used to establish the cohort. We intend to publish the detailed findings from our work in a series of related publications in due course.  * 5. "It is also a bit unclear who will be interviewed, recruited to participate in a focus group discussion, and/or surveyed." *>> In the section describing “cohort activities” we have added more detail about advertising the engagement events related to a new theme to the whole cohort, then selecting groups of students for these activities from among those who express interest, and subsequently selecting a sub-group of those who participate in an engagement event for the FGDs/IDIs relating to that theme.* *With respect to the surveys, all students in the cohort are asked to complete the demographic questionnaire and comprehensive survey as soon as possible after enrolment. At the start of each academic year all students still involved in the cohort are asked to complete the comprehensive survey again. * 6. "Does each student only have to participate in the kick-off seminar [...] especially if the study aims to determine changes in attitudes towards clinical research from participation in different activities?" *>> After recruitment (with appropriate consent), all students are encouraged to attend a kick-off seminar, though not all are able to fit this in due to their busy schedules. Later, all the students in the cohort at the time an engagement event is planned for a particular theme, are invited to express interest to take part if they wish. However, for each event numbers are limited by the venue capacity and by the number of staff available to support the event. Individual students are then selected to attend on a first come first served basis after ensuring that students who have not been able to take part previously (or recently) are first offered a place.  All activities are voluntary, and no student is “required” to attend any event. We have very active student ambassadors who encourage participation, and actually most activities are very oversubscribed.* *We plan to look at big picture changes in the students’ views by examining the serial comprehensive surveys that are completed each year. For the quantitative data, individual themes are being explored sequentially; therefore, although we may see effects related to age/year of study within a theme, we will not attempt to evaluate changes related to exposure to study activities over time. * 7. "Will the authors be comparing the impact of different kinds of activities on students’ attitudes towards/awareness of issues in clinical research?" *>> This is not included as an objective of the main project. However, in a separate piece of work we are exploring the student experience related to membership of the cohort, using a journey mapping approach after their involvement has ended. As part of this effort, we are examining the views of a small number of students about the influence of different activity types and whether they felt being involved changed their views about research in a meaningful way.  * 8. "The authors also need to describe if they have a target number of initial interviewees, focus group discussions, and number of participants in a focus group discussion." *>> The intended numbers of participants for each activity type have been added to the manuscript.  * 9. "The authors also need to indicate whether family members that will be interviewed and non-students involved in participatory initiatives will be remunerated." *>> Family members who participate in FGDs are remunerated the same standard amount for their time as the student participants.* *The student-led participatory activities are competitive in nature, and the time and effort expended by students is variable. There is no monetary benefit, but comprehensive skills training is provided for those who wish to take part, and a series of prizes is awarded to the best entries in several different categories, as judged by an independent panel. All students who submit an entry also receive a small gift of appreciation and a certificate of recognition for their participation.  * 10. "The section on Study Status should directly include some information on what counts as a “poor” or “wealthy” family. Figure 1 can also be made more comprehensive [....]  include a section indicating planned future activities (after July 31) to help readers visualize the entire project." *>> Information on how we classified socio-economic status is included in the Appendix, and we feel adding this level of detail to the main document could distract the reader. Therefore, we prefer to retain this information in the Appendix.* *Figure 1 is a summary of the events that took place during the first year, indicating the numbers of students exposed to or attending each type of event. Actually, the timing of the various activities overlaps as many events took place in parallel. We have designed a schematic (Supplementary Figure 3) to present the general flow of activities over the first year but feel it would be too confusing for the reader to include all the actual dates in Figure 1.* *Other than as described in general here, we do not have an overall plan of the entire project. Thematic activities are arranged in a flexible manner, being responsive to the interests of both the research team and student cohort members as the project progresses. * 11. "In the Discussion, the authors could describe how socio-economic status[....] how professional training (public health vs. medicine vs. nutrition) can influence understanding of ethical/societal issues in research." *>> In the Discussion section we comment on the relevance of demographic and socio-economic  characteristics and indicate that these factors can influence individuals’ attitudes towards research and research ethics. However, the intention in this paper is to describe the methodology we used to establish the cohort and the techniques we employed to explore the students’ views, and also to present a big-picture overview of the structure of the cohort after one year.  In subsequent papers we will describe our findings in relation to particular ethical concepts/questions, and at that time we will explore relationships with these, and other, factors. Regarding the potential impact of professional training, we agree that students’ perception may be influenced by knowledge gained though their academic training; when appropriate this will be included in our analyses related to particular topics. * 12. "Finally, the authors might consider double checking their punctuation[....] can be replaced with “One disease of particular concern”. *>> Grammar and syntax are continually evolving and there are differing views on many points - e.g. use of a comma after an introductory prepositional phrase. An “uncluttered” approach tends to be preferred in newer style guides. However, if the journal has an in-house style guide, we would be happy to follow its recommendations.*
